# Exploring the potential of ChatGPT in medical dialogue summarization: a study on consistency with human preferences

**DOI:** 10.1186/s12911-024-02481-8

**Published:** 2024-03-14

**Authors:** Yong Liu, Shenggen Ju, Junfeng Wang

**Affiliations:** Department of Computer Science, Sichuan University, No. 24, South Section 1, 1st Ring Road, Chendu, 610065 Sichuan China

**Keywords:** Internet Healthcare, Large language models, ChatGPT, Automated medical consultation, Medical dialogue summarization

## Abstract

**Background:**

Telemedicine has experienced rapid growth in recent years, aiming to enhance medical efficiency and reduce the workload of healthcare professionals. During the COVID-19 pandemic in 2019, it became especially crucial, enabling remote screenings and access to healthcare services while maintaining social distancing. Online consultation platforms have emerged, but the demand has strained the availability of medical professionals, directly leading to research and development in automated medical consultation. Specifically, there is a need for efficient and accurate medical dialogue summarization algorithms to condense lengthy conversations into shorter versions focused on relevant medical facts. The success of large language models like generative pre-trained transformer (GPT)-3 has recently prompted a paradigm shift in natural language processing (NLP) research. In this paper, we will explore its impact on medical dialogue summarization.

**Methods:**

We present the performance and evaluation results of two approaches on a medical dialogue dataset. The first approach is based on fine-tuned pre-trained language models, such as bert-based summarization (BERTSUM) and bidirectional auto-regressive Transformers (BART). The second approach utilizes a large language models (LLMs) GPT-3.5 with inter-context learning (ICL). Evaluation is conducted using automated metrics such as ROUGE and BERTScore.

**Results:**

In comparison to the BART and ChatGPT models, the summaries generated by the BERTSUM model not only exhibit significantly lower ROUGE and BERTScore values but also fail to pass the testing for any of the metrics in manual evaluation. On the other hand, the BART model achieved the highest ROUGE and BERTScore values among all evaluated models, surpassing ChatGPT. Its ROUGE-1, ROUGE-2, ROUGE-L, and BERTScore values were 14.94%, 53.48%, 32.84%, and 6.73% higher respectively than ChatGPT’s best results. However, in the manual evaluation by medical experts, the summaries generated by the BART model exhibit satisfactory performance only in the “Readability” metric, with less than 30% passing the manual evaluation in other metrics. When compared to the BERTSUM and BART models, the ChatGPT model was evidently more favored by human medical experts.

**Conclusion:**

On one hand, the GPT-3.5 model can manipulate the style and outcomes of medical dialogue summaries through various prompts. The generated content is not only better received than results from certain human experts but also more comprehensible, making it a promising avenue for automated medical dialogue summarization. On the other hand, automated evaluation mechanisms like ROUGE and BERTScore fall short in fully assessing the outputs of large language models like GPT-3.5. Therefore, it is necessary to research more appropriate evaluation criteria.

## Background

As healthcare evolves towards a patient-centered delivery model, online searching and accessing health information can fulfill patients’ needs for prognosis and treatment information [1, 2]. Furthermore, with the rapid development of “Internet plus Healthcare” online consultation platforms are emerging, allowing doctors to diagnose diseases and provide relevant medical advice through remote conversations with patients. On the one hand, this enhances the efficiency of the healthcare system and alleviates some of the burden on medical professionals, enabling them to invest more energy in improving patient care and minimizing time spent on irrelevant matters [3, 4]. On the other hand, it enables effective patient screening, maintains social distancing, and protects clinical doctors and communities from infections, while still offering personalized healthcare and medical services [5]. During the COVID-19 pandemic, influenced by policies and the pandemic’s impact, the demand for online consultations has rapidly increased. This has irrevocably altered the status of telemedicine in the U.S. healthcare system and has been widely adopted across global healthcare systems [6]. Summarizing conversations on remote medical platforms can bring about several benefits. For instance, Both doctors and patients can refer to important parts or conclusions from past interactions. This not only allows patients to quickly access the results they are concerned about but also enables doctors to learn from the experiences and approaches of other medical professionals when dealing with similar issues. Medical text summarization algorithms related to medical dialogue summaries are techniques that automatically extract key information from various medical data sources such as medical literature, electronic health records, and medical dialogue, and generate concise summaries. These algorithms mainly include two types: extractive summarization and abstractive summarization.

Extractive summarization selects content based on importance or keywords in the text. The generation process does not involve creating new sentences or phrases; it simply selects and combines existing content from the original text to generate a summary. It often treats summarization as a sequence labeling task, where each sentence is labeled with a binary classification tag of “yes” or “no”, and the summarization process can be viewed as the selection of sentence classification labels. BERTSUM is a text summarization model based on bidirectional encoder representation from Transformers (BERT). It aims to leverage the powerful representation capabilities of BERT to generate concise summaries from input text [7]. A hierarchical encoder-tagger model enhanced with a memory module was proposed to identify important utterances in dialogues between patients and doctors, thereby accomplishing the task of medical dialogue summarization [8]. However, this method selects essential sentences from the original text to form concise summaries, maintaining interpretability and accuracy, however, it lacks the ability to generate new sentences, potentially resulting in less smooth and coherent summaries [8].

The abstractive summary uses natural language generation techniques to create new sentences or phrases by understanding the semantics and context of the original text in order to generate a summary. Abstractive summarization can typically express information more freely, rather than relying solely on the extraction of content from the original text. Kundan et al. [9] proposed a bidirectional LSTM-based encoder-decoder model with attention. It is used to extract important phrases related to each section of the summary and concatenate these relevant phrases together to generate a summary sentence for each cluster. The BART model combines the encoder-decoder architecture of Transformer with a pre-training task involving denoising autoencoders. This structure helps the model capture complex relationships between input data in text generation tasks [10]. A simple yet general two-stage fine-tuning method is proposed to deal with input length limitations of the model, enabling the step-by-step generation of medical dialogue summaries [11]. George et al. [12] proposed a sequence-to-sequence architecture for summarizing medical dialogues by integrating medical domain knowledge from the Unified Medical Language System (UMLS). Medical concepts from the references are encoded to distinguish important medical concepts, and combining an end-to-end approach to explicitly model a switching variable, induce a mixed model of copying, generating, and negation to obtain medical dialogue summaries [13]. The abstractive summary algorithm uses deep learning techniques such as RNN or Transformer to generate new, fluent, and coherent summaries, and ensures accuracy while avoiding the generation of unreasonable summaries when dealing with complex medical texts [11, 14]. The success of Transformer is attributed to their high degree of parallelism and self-attention mechanism. Building upon this foundation, the BERT model improved the architecture and achieved universal language representations through unsupervised pre-training on large-scale corpora [15]. This study inspired a great deal of subsequent work, establishing the “pre-training and fine-tuning” learning paradigm, and introducing different architectures such as BART [16] and GPT-2 [17] that can be used to fine-tune downstream tasks. As the scale of parameters continues to increase to hundreds of billions, and training on massive data, large language models such as GPT are eventually generated, including GPT-3 and GPT-4 [18].

In recent years, LLMs provides opportunities for instructional fine-tuning through reinforcement learning from human feedback (RLHF) [19] to adapt to a variety of NLP tasks while aligning the model with human intent. They excel in areas such as education, healthcare, text generation and human-computer interaction. Summarization based on LLMs employs ICL and pre-trained language models (PLMs) to generate clinical notes, the results show that the ICL-based approach is as well-received as human-written notes. This makes it a promising approach for automatically generating notes in medical dialogues [20]. Expert validation demonstrates that clinical notes generated by ICL in GPT-4 outperform all traditional fine-tuned models [21]. The applicability of large language models in radiology report summarization tasks was explored by optimizing input prompts based on a small number of existing samples and an iterative approach [22]. ChatGPT allows doctors to input specific information and medical concepts related to patients and generate formal patient discharge summaries. Automating this process can reduce the workload of junior doctors, giving them more time to provide patient care and seek training opportunities [23].

**Fig. 1:**
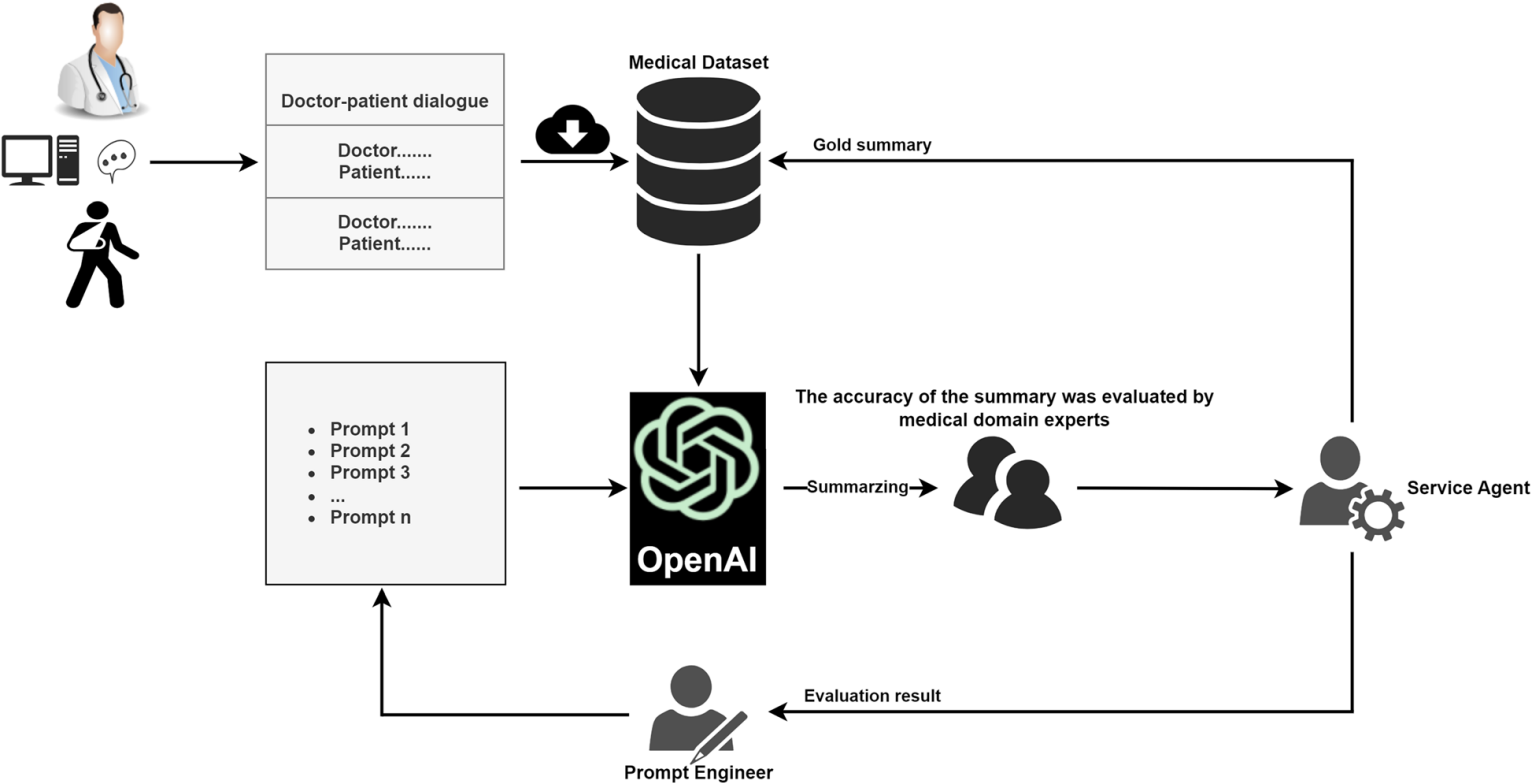
The processing flow of generating summaries from doctor-patient dialogues using ChatGPT

**Fig. 2:**
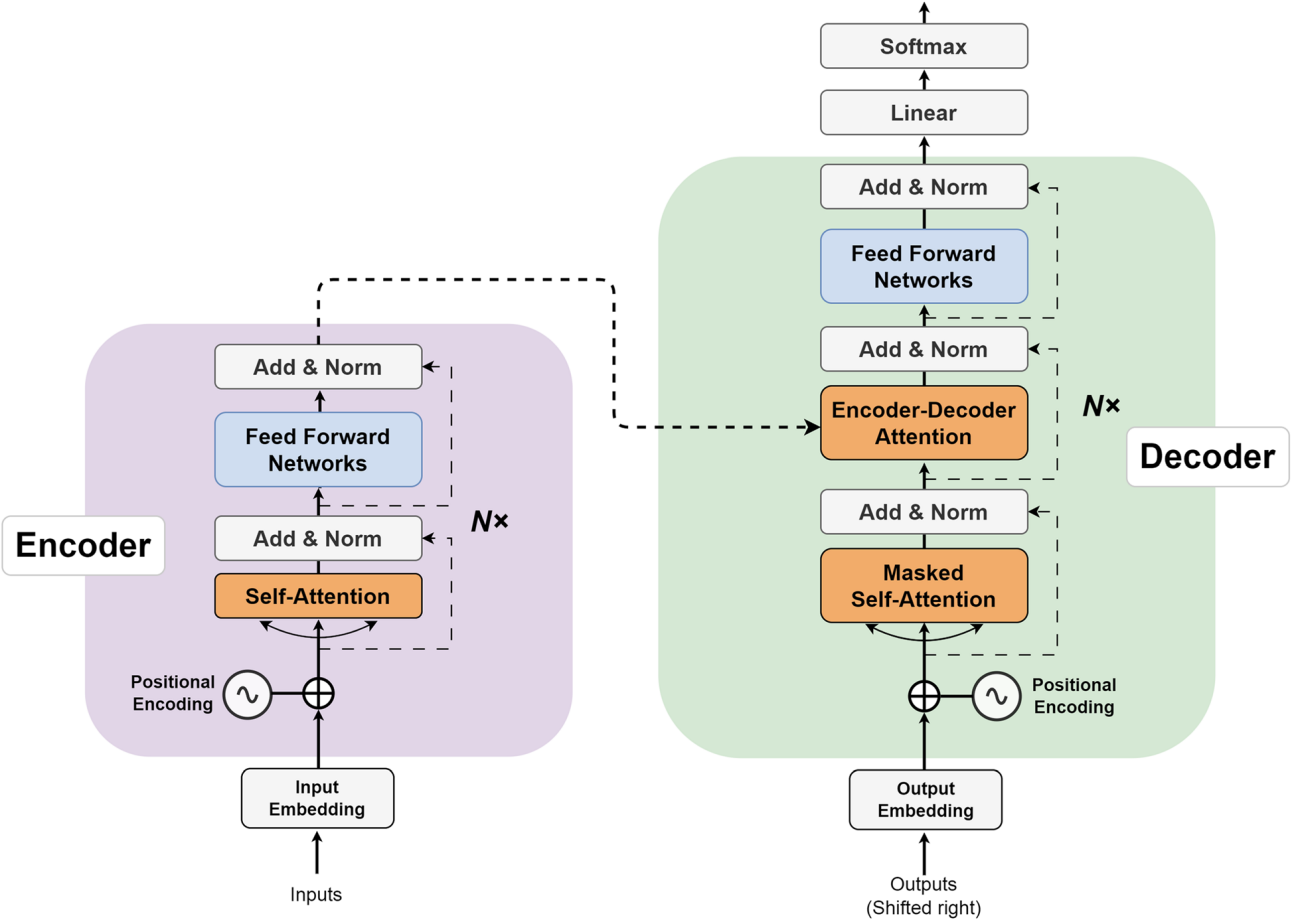
The Transformer - model architecture. The creation of this figure is based on Fig. 1 in the paper [25]

In this paper, We utilized two different approaches to generate medical dialogue summaries on a publicly available medical conversation dataset. Method A involves fine-tuning PLMs such as BERTSUM [7] and BART [16], while Method B utilizes the large language model ChatGPT based on ICL [24], whose processing flow is shown in Fig. 1. For Method B, we first fine-tune the relevant parameters of the ChatGPT model and then explore using the model’s prompt engineering functionality to generate medical dialogue summaries, and finally measure the ability of ChatGPT to generate medical dialogue summaries by automatic and human evaluation criteria.

## Methods

### Model

#### Transformer

The “Transformer” model has become a foundational deep learning architecture for natural language processing [25]. As shown in Fig. 2, the Transformer model is composed of encoder and decoder layers. Each of the encoder layer has a multi-head self-focusing and position-wise feed-forward network (FFN) sub-layer. The multi-head attention mechanism utilizes parallel scaled dot-product attention functions to focus on different subspaces and positions in the input data. By calculating the attention between query (Q), keys (K), and values (V), the results are as follows:

1 $$\begin{aligned} Attention\left( Q,K,V \right) = softmax\left( \frac{QK^{T}}{d_{k} } \right) V \end{aligned}$$

In Eq. (1), a set of queries , keys and values are packed together into a matrices *Q*, *K* and *V*. In addition, ${d_{k}}$ usually refers to the dimension of the key vector, which can be used as a scaling factor to avoid excessive dot products. ReLU activation is used in the FFN sub-layer. In addition, layer normalization and a residual connection link the two sub-layers and can be used to tackle gradient issues , thus, stable network training can be obtained. Each decoder layer includes three sub-layers: an FFN sub-layer and two attention sub-layers. The decoder self-attention sub-layer uses a mask function to prevent attending to unseen future tokens. The encoder-decoder attention layer enables the decoder to focus on essential parts of the source sequence and capture the encoder-decoder relationship.

Given an input sequence of symbol representations $X = [x_{1}, x_{2},..., {x_{n}}]$ and a real output sequence $Y = [{y_{1}}, {y_{2}},..., {y_{m}}]$. We assume that the last position of each input sequence is a special “[END]” flag. The encoder maps *X* to a sequence of continuous representations $H = [h_{1}, h_{2},..., {h_{n}}]$. Given *H*, the decoder then generates a predicted output sequence $\hat{Y} = \left[ \hat{y_{1} },\hat{y_{2}},...,\hat{y_{k}} \right]$. Therefore, during training, the model minimizes the cross-entropy loss between the predicted sequence $\hat{Y}$ and the real output sequence *Y*, as shown in Eq. (2), where *k* represents the number of target sequences. At each step, the model is auto-regressive [26], which means that it utilizes the previously generated symbols as additional input when generating the next symbol in the sequence. This enables the model to contextually understand and produce coherent outputs in a step-by-step manner.

2 $$\begin{aligned} L\left( \hat{Y},Y \right) = -\sum \limits _{i=1}^{m} \hat{y}_{i} log {y}_{i} \end{aligned}$$

The Transformer’s impact on natural language processing has been profound, inspiring the development of modern NLP models such as BERT [15], GPT [17], RoBERTa [27], and T5 [28], all built on the Transformer architecture.

#### BERT

**Fig. 3:**
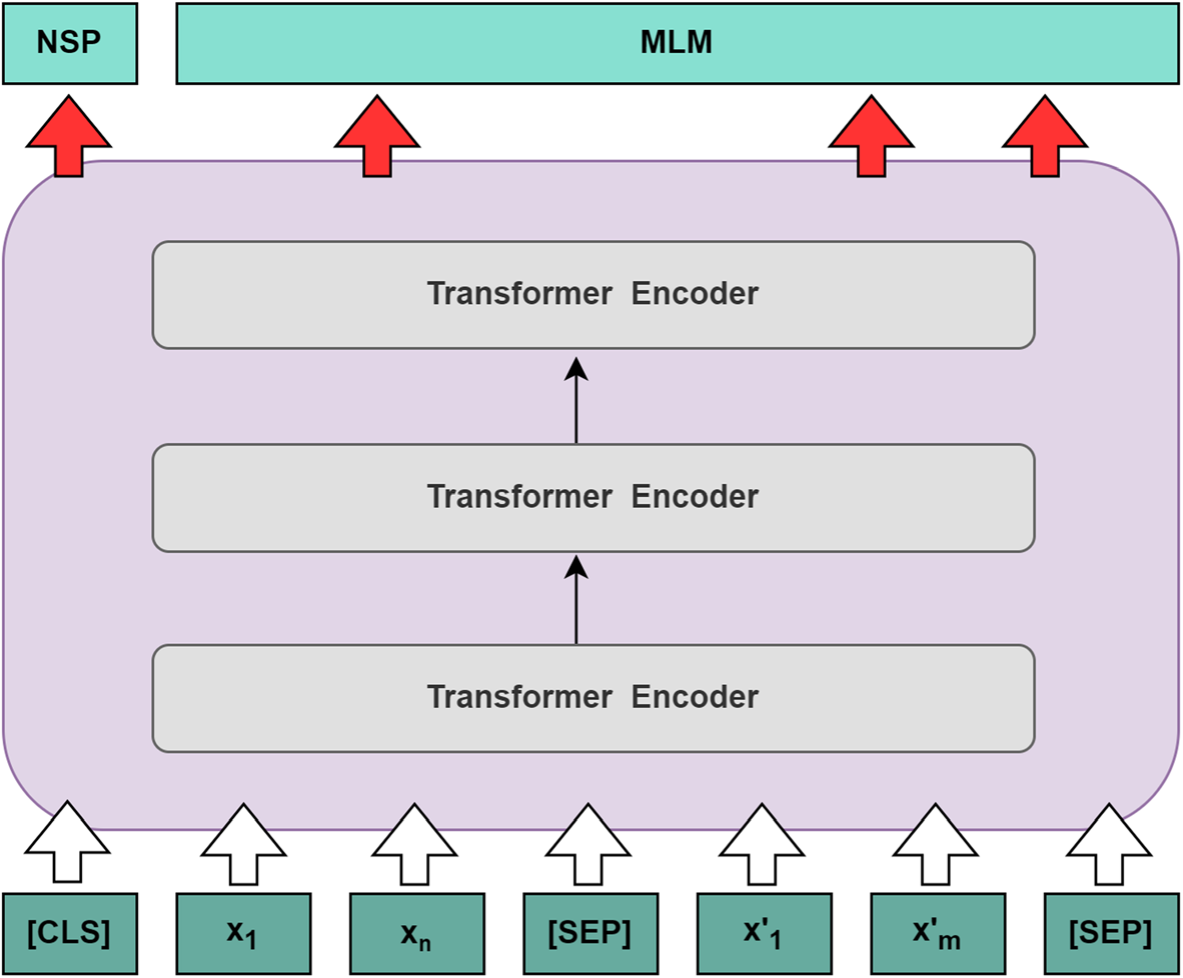
The overview architecture of the BERT model. The creation of this figure is based on Fig. 1 in the paper [15]

BERT is an important language model based on the Transformer architecture, it is trained to learn general language representations and capture contextual semantics, which has had a profound impact on NLP research and applications [29–31]. As shown in Fig. 3, the basic BERT structure is made up of multiple layers of Transformer and includes two pre-training tasks: mask language model (MLM) and next sentence prediction (NSP). Taking NSP as an example, the main elements are divided three parts as follows:

- Input layer. For a given input text that has undergone masking and is represented as $x = [x_{1}x_{2}...x_{n}]$ and $x ^{'} = [x ^{'}_{1}x ^{'}_{2}...x ^{'}_{m}]$ , the following processing results in the BERT input representation *e*.
  3 $$\begin{aligned} X = [CLS]x_{1}x_{2}...x_{n}[SEP]x ^{'}_{1}x ^{'}_{2}...x ^{'}_{m}[SEP] \end{aligned}$$
  4 $$\begin{aligned} e = InputRepresentation\ \left( X \right) \end{aligned}$$
  In Eq. (3), [CLS] represents the special token marking the beginning of a text sequence, and [SEP] represents a separator marker between text sequences.
- BERT encoder layer. In this layer, the input representation *e* is encoded by *L* layers Transformer to obtain a contextual semantic representation of the input text:
  5 $$\begin{aligned} h = Transformer\ \left( e \right) \end{aligned}$$
  where $h \in \mathbb R ^{N\times d }$, *N* represents the maximum length of the sequence, and *d* represents the hidden layer dimension of BERT.
- The output layer. In the NSP task, BERT uses the hidden layer of the [CLS] position as the semantic representation of the context consisting of the first component $h _{0}$ of *h*, and finally predicts the classification probability *P* of the input text through a fully connected layer:
  6 $$\begin{aligned} P = Softmax\left( h _{0}W^{p} + b^{0} \right) \end{aligned}$$
  where $P \in \mathbb R ^ {2}$, $W^{p} \in \mathbb R ^{d\times 2 }$ represents the weight of the fully connected layer, and $b^{0} \in \mathbb R ^ {2}$ represents the bias of the fully connected layer. Classification probability *P* is used to calculate cross-entropy loss with the real label *y*. The final model parameters are updated based on this loss.

The application of BERT in medical document summarization accelerates the acquisition [32, 33], processing and application of medical information [34, 35], improving the efficiency and accuracy of healthcare and medical research [36]. It brings a broader development space for the medical field, and is expected to promote the innovation of medical information processing and application in the future [37].

#### BERTSUM

BERTSUM is a text summarization model based on BERT which aims to generate concise summaries from input text by leveraging BERT’s powerful representation capabilities. The model utilizes BERT’s encoder to obtain semantic vector representations for sentence-level segments in the input text. The training of BERTSUM occurs in two stages: pre-training of the BERT model on unsupervised tasks and supervised fine-tuning with datasets containing human summaries. During fine-tuning, the model optimizes the similarity between generated and original summaries as the loss function [7].

**Fig. 4:**
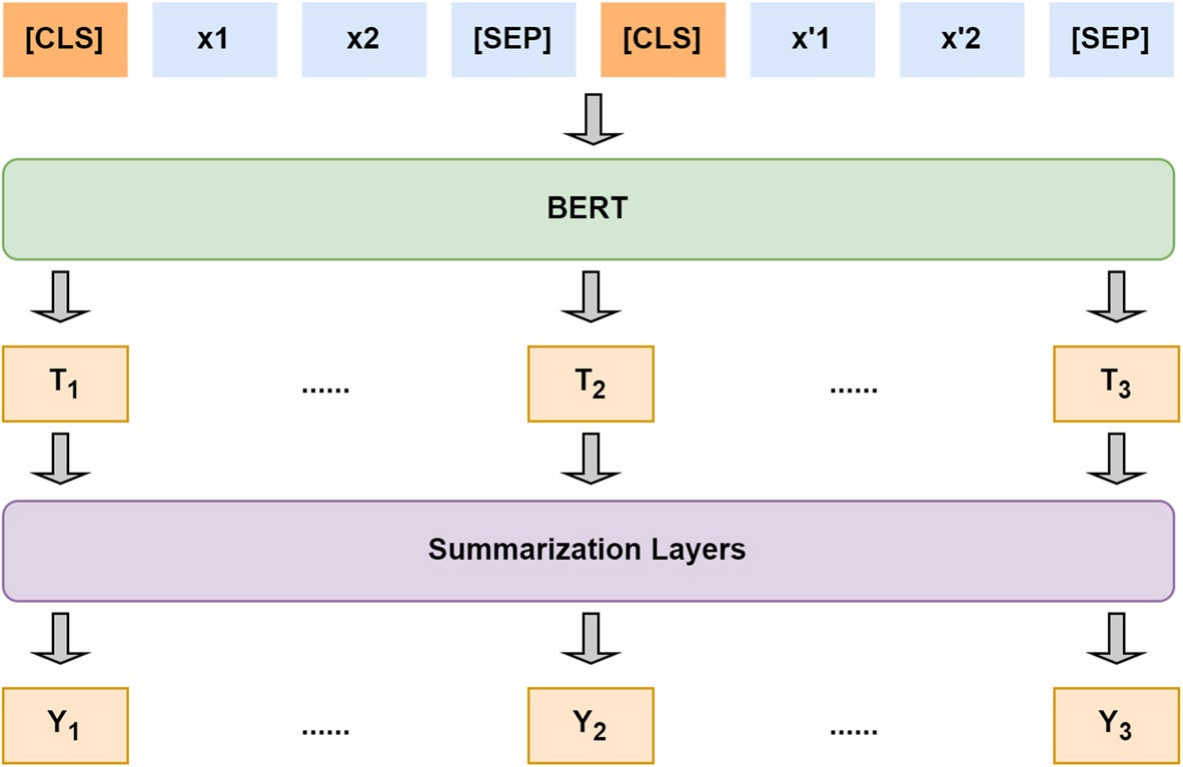
The overview architecture of the BERTSUM model. The creation of this figure is based on Fig. 1 in the paper [7]

As shown in Fig. 4, in the context of encoding multiple sentences, an [CLS] token is inserted before each sentence, and a [SEP] token is inserted at the end of each sentence. In vanilla BERT, [CLS] is used to aggregate features from a single sentence or a pair of sentences. Therefore, by using multiple [CLS] tokens to fine-tune the model and based on these tokens, it is possible to obtain sentence features in ascending order. The BERT sentence vectors undergo additional summarization-specific layers to capture document-level features for summary extraction. The resulting values are then passed through the sigmoid function, which maps them to a range between 0 and 1. Therefore, each sentence is assigned a predicted score $\hat{Y}$.

7 $$\begin{aligned} \hat{Y} = \sigma \left( W_{o} T_{i} + b_{o} \right) \end{aligned}$$

The model’s loss is the binary classification entropy between $\hat{Y}$ and the gold label Y.

#### BART

**Fig. 5:**
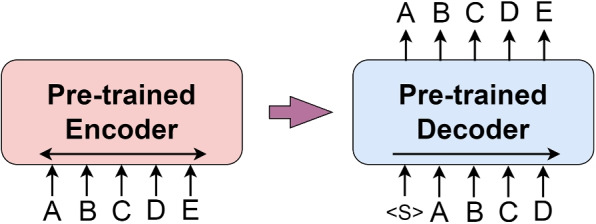
Example of a task used by the BART model for text generation. The creation of this figure is based on Fig. 3 in the paper [16]

BART is an NLP pre-trained model proposed by Facebook AI for pre-training the bidirectional and auto-regressive combined model Transformers [16]. The main idea is to combine the encoder-decoder structure of Transformer with the pre-training task of denoising auto-encoder. BART uses an encoder-decoder structure similar to Transformer. The encoder is responsible for mapping the input sequence to an intermediate representation, and the decoder then maps this intermediate representation back to the original input space. This structure can help the model capture complex relationships between inputs. In the text generation task, the input of the encoder is the input text as the condition, and the decoder generates the corresponding target text in an auto-regressive way, as shown in Fig. 5.

The BART model has acquired a substantial amount of basic language knowledge during the pre-training phase, so during downstream tasks (such as text classification, named entity recognition, question answering system, document summarization, etc.), we only need to fine-tune the model, without needing to train the model from scratch. This greatly saves training time and improves the performance of the model.

**Fig. 6:**
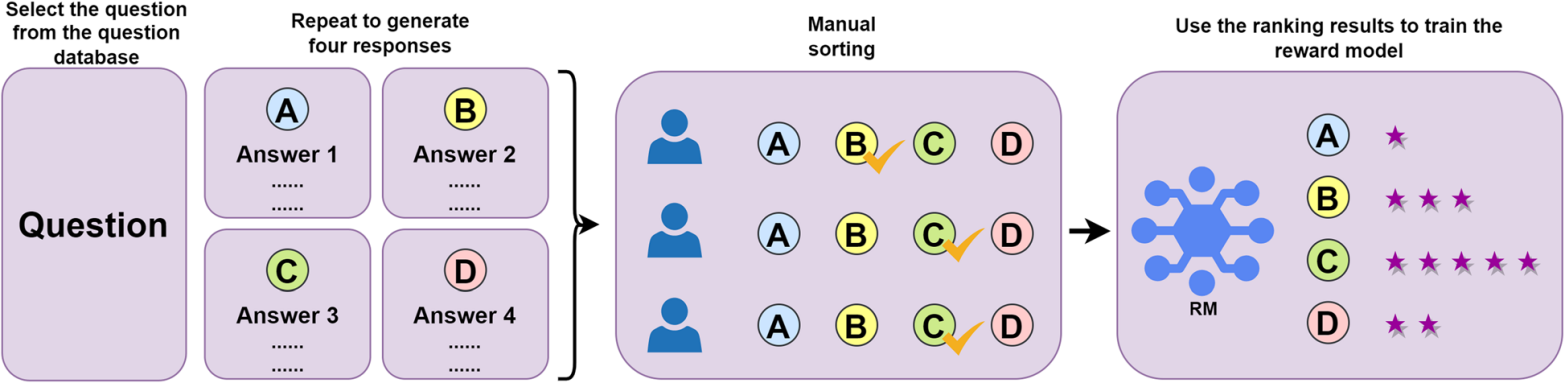
Training Process of the reward model (RM)

### ChatGPT and prompt

#### ChatGPT

ChatGPT is a powerful language model developed by OpenAI based on the Transformer architecture, which can be implemented in three steps [19]. Step 1, Collect demonstration data and train a supervised policy. Step 2, Collect comparison data and train a reward model. Step 3, Optimize the reward model using proximal policy optimization (PPO) [38]. Figure 6 illustrates the relevant process of Step 2, where annotators annotate the data in the candidate dataset according to their respective standards and manually rank them based on the scores. Then, they input the rankings into a reward model to predict the preferences of the manual annotations.

The loss function used during training is shown in formula (8).

8 $$\begin{aligned} Loss\left( \theta \right){} & {} = -\frac{1}{\left( \genfrac{}{}{0.0pt}0{K}{2} \right) } E\left( x,y_{w},y_{l} \right) \nonumber \\{} & {} \sim D \left[ log\left( \sigma \left( r_{\theta } \left( x,y_{w} \right) -r_{\theta }\left( x,y_{l} \right) \right) \right) \right] \end{aligned}$$

where $r_{\theta }\left( x,y \right)$ is the scalar output of the reward model for prompt *x* and completion *y* with parameters $\theta$ , $y_{w}$ is the preferred completion out of the pair of $y_{w}$ and $y_{l}$, and *D* is the comparison dataset.

As a generative model, ChatGPT can produce text sequences given an initial prompt. It comes in different versions, with each new iteration being more capable and better at handling complex language tasks [39]. ChatGPT finds applications in various fields [40], including tutoring and education [41], translation [42], healthcare [43], and medicine [44–46].

Regarding medical text summarization, users can utilize ChatGPT to understand and condense lengthy medical reports or research papers [47]. By using a relevant portion of the text as a prompt, the model can provide a concise summary tailored to the user’s needs [48]. For example, a user studying heart disease could prompt the model to generate a simplified summary of coronary artery disease and its causes.

#### Prompts for large language models

In ChatGPT or other GPT models, “prompt” refers to the initial text or request entered into the model. This can be a question, part of a sentence, or even a word. The model generates or continues text based on this initial prompt, with the goal of producing fast responses that are consistent in terms of grammar, context, and style. It achieves this by relying on the language patterns and associations learned during the pre-training phase, as well as the more specific guidance acquired during the fine-tuning phase.

By optimizing the design of prompt, users can better guide models to produce outputs that meet their specific needs. We try to provide some reference content as follows:

- Define the task or objective. Clearly specify the task or objective you want the model to accomplish. This could be answering questions, generating articles,etc.
- Understand the model’s capabilities. Familiarize yourself with the limitations and strengths of the model. Different models perform differently on tasks, and understanding their capabilities helps guide them effectively.
- Choose appropriate length and format. Determine the length and format of the prompt. Sometimes, concise prompts are more effective, but for certain tasks, a more detailed description or context may be necessary.
- Grammar and format. Ensure the prompt is grammatically correct and adheres to the input format expected by the model. This increases the likelihood of the model understanding and generating correct output.
- Provide relevant information. If specific background or contextual information is required, make sure to include it in the prompt. This helps the model better understand the task or question.
- Iterate for optimization. Iterate through different prompts, observe the model’s responses, and adjust based on the results. This is an iterative process that gradually improves the model’s performance.
- Evaluate and adjust. Assess whether the generated text aligns with expectations and adjust or improve prompts as needed. Continuous evaluation of outputs guides the optimization process.

Follow the above description, users can better guide models to produce outputs that meet their specific needs. Overall, prompt is an important factor in driving GPT models to produce specific outputs, and users can carefully design prompt to get the best model output [49].

### Experiments

#### Dataset

**Fig. 7:**
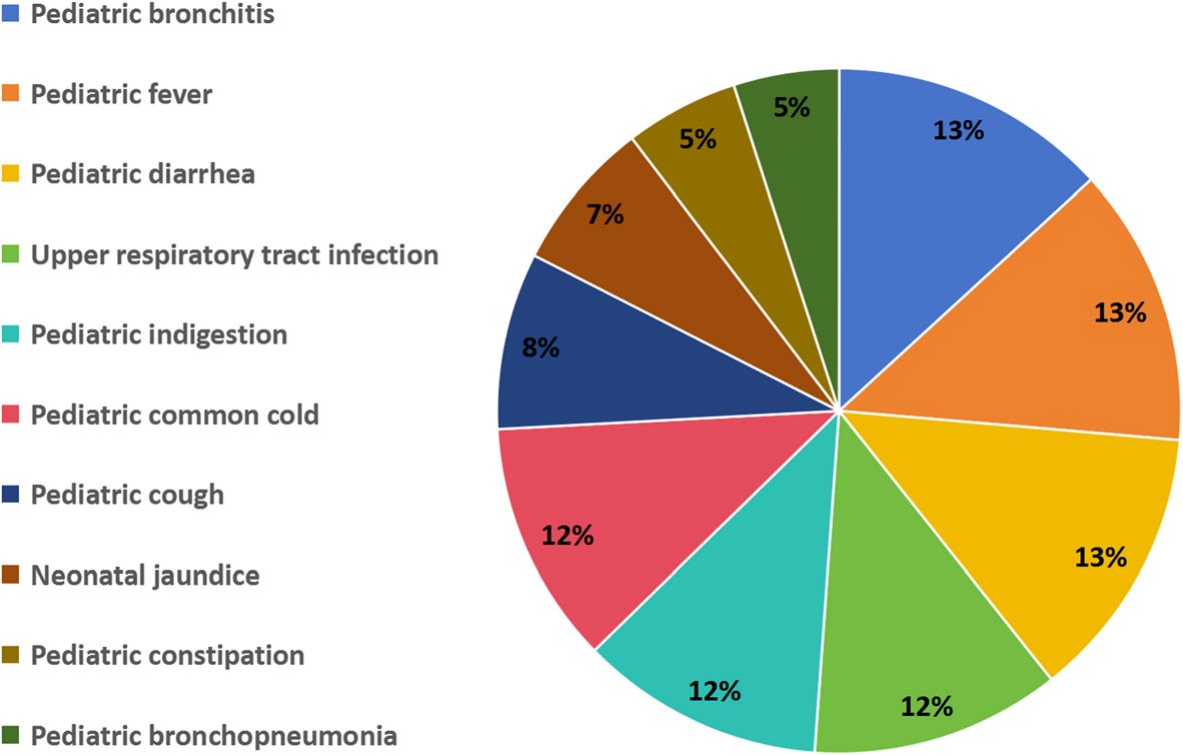
The proportion distribution of the 10 pediatric diseases in the IMCS-V2 medical dialogue dataset

We utilized the data provided by the School of Data Science at Fudan University, which was constructed under the guidance of medical experts from Fudan University Medical School and named IMCS-V2. This dataset has collected authentic online medical dialogues and subjected them to multi-level human annotations. The aim is to facilitate open evaluation against the Chinese biomedical language understanding evaluation (CBLUE) benchmark and thereby advance the fields of intelligent healthcare and medical language understanding. The IMCS-V2 dataset comprises 4,116 medical-patient dialogue samples that have undergone meticulous annotations, and detailed statistical data are presented in the following Table 1. In addition, this dataset encompasses 10 pediatric diseases, and the disease distribution is shown in Fig. 7.

**Table 1:** Detailed statistics on the IMCS-V2 medical dialogue dataset

Statistical indicators	Value
Total Diseases	10
Total Dialogues	4116
Total Sentences	164731
Average Sentences/Per Dialog	40
Average Words/Per Dialog	523

The dataset used in this paper is a publicly available dataset designed for a medical natural language processing competition. The task involves generating medical reports from multi-turn doctor-patient dialogues. However, due to the competition’s requirements, the summary portions of the test set have been omitted. This limitation prevents us from comparing it with the summaries generated by ChatGPT. Therefore, only (4116 - 811)=3305 samples out of the original 4116 samples are available for the final training and testing. We migrate the first 500 samples (sorted by file name) from the valid set as the new test set, and then combine the first 167 samples from the training set (sorted by file name) with the remaining samples from the valid dataset to form a new valid dataset consisting of 500 samples. In the end, train set has 2305 samples, valid and test sets have 500 samples respectively. Since the samples distribution in the original train and valid sets were randomly generated by the organizer, we did not carry out random selection in the process of data migration, but only extracted according to the order of file names.

#### Experimental environment and model parameter settings

In this paper, all the experiments were conducted on two NVIDIA GeForce RTX 3090 GPUs with 24GB of memory and using the python language on the PyCharm platform. The relevant environment settings required for the experiment are shown in Table 2. The data is divided into a training set of 2305, a verification set of 500 and a test set of 500. In this paper, abstracts are divided into two types: extractive summarization and abstractive summarization. BERTSUM model is the representative of extractive summarization, while BART model and ChatGPT are the representatives of abstractive summarization. The training parameters of BERTSUM model are shown in Table 3, and the training parameters of BART model are shown in Table 4.

**Table 2:** Hardware and software environment

Device	Congiguration
Operating system	Ubuntu 20.04.6 LTS
Processor	Intel Xeon(R) Gold6133 CPU ®2.50GHz
GPU	RTX 3090 (24GB)*2
Framework	Pytorch
Compilers	PyCharm
Scripting language	Python 3.8

**Table 3:** Hyper-parameters of BERTSUM, in the case of multiple candidate parameter values, the ultimately chosen parameter value is displayed in bold

Parameters	Values
encoder	(**classifier**/transformer/rnn)
batch size	(1000/2000/**3000**)
train steps	10,000
dropout	0.1
learning rate	$2e^{-3} \cdot min\left( step^{-0.5}, step \cdot warmup^{-1.5} \right)$
warmup	(**1000**/10,000)
optimizer	adam

**Table 4:** Hyper-parameters of BART

Parameters	Values
batch_size	32
epochs	3
max_input_length	521
max_target_length	150
learning_rate	1e-04
warmup_steps	10
weight_decay	0.001
metric_for_best_model	ROUGE-1

**Fig. 8:**
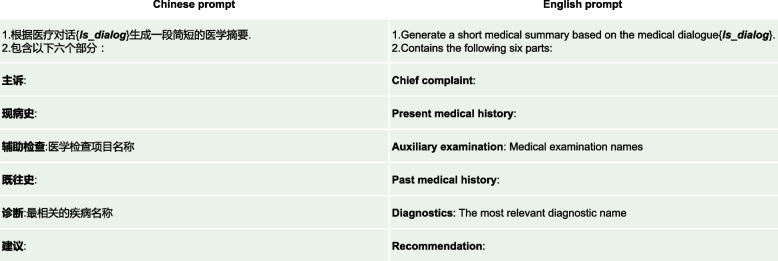
A simple prompt for medical dialogue summarization without any complex parameter variables, abbreviated as Prompt_S

**Fig. 9:**
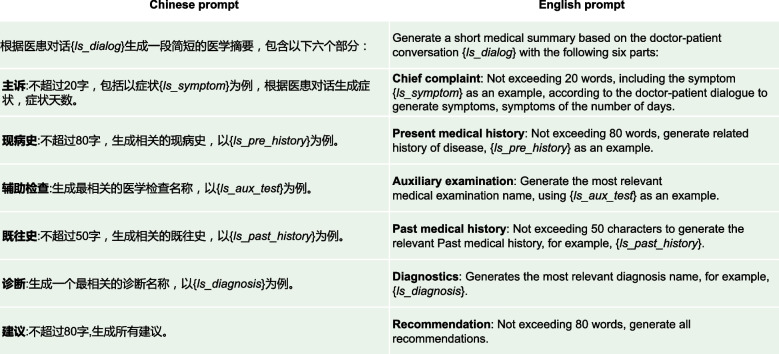
A technical prompt for medical dialogue summarization with some parameter variables, abbreviated as prompt_T

**Fig. 10:**
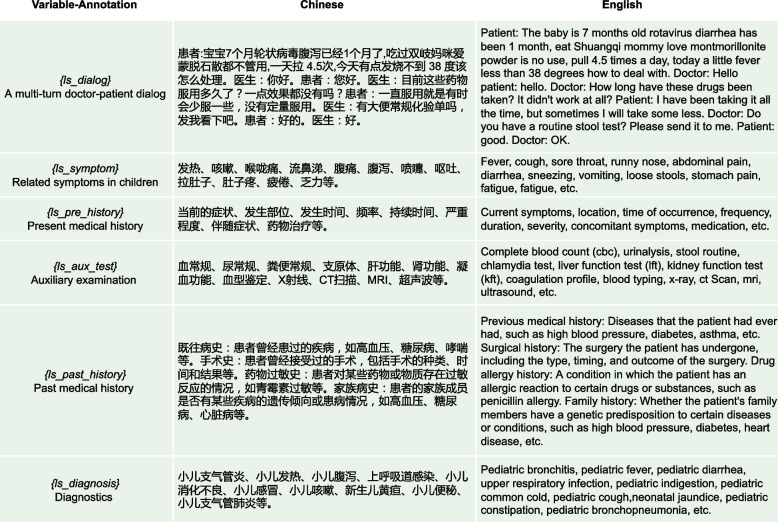
Detailed description of the parameters related to prompt_T

#### Prompt settings for summarization

Figures 8, 9 and 10 show prompt settings related to ChatGPT model. The prompts in this paper are divided into two types: simple type, denoted as Prompt_S, as shown in Fig. 8, and technical type, denoted as Prompt_T, as shown in Fig. 9. In Fig. 8, Prompt_S does not use complicated prompts, but indicates “Medical examination names” in the “Auxiliary examination” part, and “The most relevant diagnostic name” in the “Diagnosis” part, in the hope that ChatGPT can return relevant results.

Since this article is based on research using a Chinese medical dialogue dataset, the prompts used by ChatGPT are written in Chinese. Actually, ChatGPT itself supports multiple languages, so you can also write prompts in English, and simply include an additional instruction for ChatGPT to provide medical dialogue summaries in Chinese. Please note that there will be several instances of Chinese content in this text, along with corresponding English explanations, just to make it clearer for readers who use different languages. In Fig. 9, taking advantage of the idea of task decomposition, we use Prompt_T to split the summary into six parts, each with related more detailed sub-prompts. For example, the “Chief complaint: Not exceeding 20 words, including symptoms such as {*ls_symptom*} as an example, generated based on the doctor-patient dialogue, and the duration of symptoms.”. Symptoms are stored in the variable {*ls_symptom*}, which mainly refers to symptoms of childhood diseases related to the dataset, including “fever”, “cough”, “sore throat”, “runny nose”, “abdominal pain”, “diarrhea”, etc. Please refer to Fig. 10 for detailed explanations. The purpose of this approach is to try to give ChatGPT, in this way, a contextual example of similar symptoms of the disease, so that it can give a better description of the patient’s symptoms. Similarly, in the “Auxiliary examination” part, “generate the most relevant medical examination names, using {*ls_aux_test*} as an example”. The variable {*ls_aux_test*} stores some medical examination names, such as “complete blood count”, “urinalysis”, “stool routine”, “chlamydia”, “liver function”, “kidney function”, etc. Eventually, ChatGPT is expected to give a better name for the auxiliary examinations performed by the patient based on the content of this variable {*ls_aux_test*}. For the rest of Fig. 9, some give specific examples, such as the “Diagnosis” part, and some give relevant explanations, such as the “Present medical history” part. For details, please refer to the corresponding contents in Fig. 10, which will not be repeated here.

### Evaluation metrics

#### Automatic evaluation metrics

In this paper, the ROUGE-1, ROUGE-2, ROUGE-L [50] and BERTScore [51] as automatic evaluation metrics.

Given a reference summary $x=\left( x_{1}, \dots , x_{k} \right)$ and a candidate summary $\hat{x} =\left( \hat{x} _{1}, \dots , \hat{x} _{k} \right)$. By embedding model generate reference summary vector $X=\left( X_{1}, \dots , X_{m} \right)$ and candidate summary vector $\hat{X} =\left( \hat{X} _{1}, \dots , \hat{\mathrm {X}} _{m} \right)$, respectively.

ROUGE-n represents an n-gram recall measure comparing a reference summary to the corresponding candidate summary. The computation of ROUGE-n is as follows:

9 $$\begin{aligned}{} & {} P_{ROUGE-n} = \frac{Count _{match}\left( gram_{n} \in \left( x,\hat{x} \right) \right) }{Count \left( gram_{n} \in \hat{x} \right) } \nonumber \\{} & {} R_{ROUGE-n} = \frac{Count _{match}\left( gram_{n} \in \left( x,\hat{x} \right) \right) }{Count \left( gram_{n} \in x \right) } \nonumber \\{} & {} F_{ROUGE-n} = \frac{2*P_{ROUGE-n}*R_{ROUGE-n}}{P_{ROUGE-n}+R_{ROUGE-n}} \end{aligned}$$

Where *n* denotes the length of the n-gram, so $gram_{n}$ represents the 1-gram or 2-gram, and $Count _{match}\left( gram_{n} \in \left( x,\hat{x} \right) \right)$ signifies the maximum number of 1-gram or 2-gram co-occurring in a reference summary and the corresponding candidate summary.

We known that the longer the Longest Common Subsequence (LCS) of two summary sentences, the more similar the two summaries are. We employ the ROUGE-L to measure the similarity between a reference summary and the corresponding candidate summary. The calculation is as follows:

10 $$\begin{aligned}{} & {} P_{ROUGE-L} = \frac{LCS\left( x,\hat{x}\right) }{\left| \hat{x} \right| } \nonumber \\{} & {} R_{ROUGE-L} = \frac{LCS\left( x,\hat{x}\right) }{\left| x \right| } \nonumber \\{} & {} F_{ROUGE-L} = \frac{2*P_{ROUGE-L}*R_{ROUGE-L}}{P_{ROUGE-L}+R_{ROUGE-L}} \end{aligned}$$

Where $LCS\left( x,\hat{x}\right)$ is the length of a longest common subsequence of the reference summary and the corresponding candidate summary. $\left| {x} \right|$ is the length of the reference summary and $\left| \hat{x} \right|$ is the length of the candidate summary.

BERTScore computes the complete score by matching each token in candidate summary $\hat{x}$ to a token in reference summary *x* to calculate precision, and each token in reference summary *x* to a token in candidate summary $\hat{x}$ to calculate recall .

11 $$\begin{aligned}{} & {} \ COS \left( X_{i},\hat{X}_{j} \right) = \frac{X_{i}^{T}\hat{X}_{j}}{\left\| X_{i} \right\| \left\| \hat{X_{j} } \right\| } \nonumber \\{} & {} P_{BERT} = \frac{1}{\left| \hat{x} \right| }\sum \limits _{\hat{x} _{j}\in \hat{x} } \max _{x_{i}\in x} \left( \ COS \left( X_{i},\hat{X}_{j} \right) \right) \nonumber \\{} & {} R_{BERT} = \frac{1}{\left| x \right| }\sum \limits _{x_{i}\in x } \max _{\hat{x}_{j}\in \hat{x}} \left( \ COS \left( X_{i},\hat{X}_{j} \right) \right) \nonumber \\{} & {} F_{BERT} = \frac{2* P_{BERT}*R_{BERT} }{ P_{BERT}+R_{BERT} } \end{aligned}$$

Where $COS \left( X_{i},\hat{X}_{j} \right)$ is the cosine similarity of a reference summary *x* and a candidate summary $\hat{x}$ is given by the formula $\frac{X_{i}^{T}\hat{X}_{j}}{\left\| X_{i} \right\| \left\| \hat{X_{j} } \right\| }$. $\left| {x} \right|$ is the length of the reference summary and $\left| \hat{x} \right|$ is the length of the candidate summary.

**Table 5:** Temperature and Top_p parameter combinations for ChatGPT’s prompt model, such as Prompt_S and Prompt_T

Prompt Model	Temperature	Top_p
Prompt_S.1	1.0	1.0
Prompt_S.2	0.7	1.0
Prompt_S.3	0.1	1.0
Prompt_S.4	0.5	0.5
Prompt_S.5	1.0	0.1
Prompt_S.6	0.7	0.1
Prompt_S.7	0.1	0.1
Prompt_T.1	1.0	1.0
Prompt_T.2	0.7	1.0
Prompt_T.3	0.1	1.0
Prompt_T.4	0.5	0.5
Prompt_T.5	1.0	0.1
Prompt_T.6	0.7	0.1
Prompt_T.7	0.1	0.1

**Table 6:** Rouge and BERTScore scores for ChatGPT’s prompt model, such as Prompt_S and Prompt_T

Prompt Model	ROUGE-1	ROUGE-2	ROUGE-L	BERTScore
Prompt_S.1	43.69	21.74	35.78	71.43
Prompt_S.2	44.09	22.01	36.54	71.47
Prompt_S.3	43.92	21.69	36.59	71.31
Prompt_S.4	44.17	21.84	36.67	71.42
Prompt_S.5	44.18	21.88	36.87	71.40
Prompt_S.6	44.28	21.94	36.89	71.48
Prompt_S.7	44.27	21.95	36.91	71.49
Prompt_T.1	47.21	25.83	39.41	73.34
Prompt_T.2	47.84	**26.31**	40.35	73.38
Prompt_T.3	47.97	25.46	40.59	73.32
Prompt_T.4	48.07	25.50	40.57	73.36
Prompt_T.5	48.11	25.40	40.73	73.37
Prompt_T.6	48.07	25.39	40.69	73.37
**Prompt_T.7**	**48.19**	25.41	**40.81**	**73.38**

**Fig. 11:**
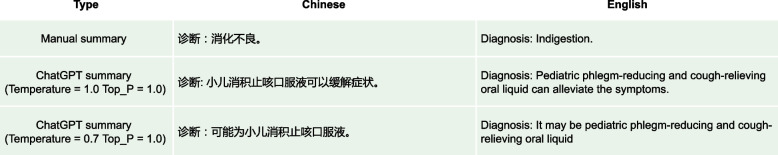
Higher values of the Temperature and Top_P parameters may lead to partial summary results generated by ChatGPT that may be inconsistent with the actual situation

**Fig. 12:**
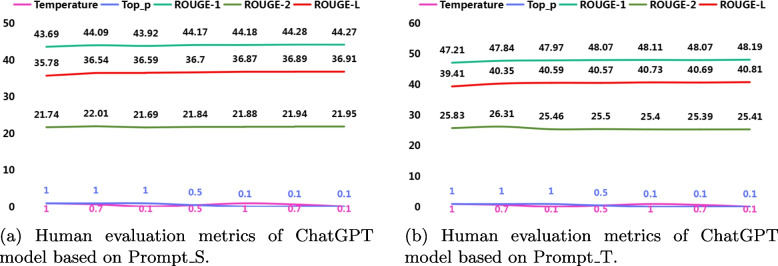
The “Temperature” parameter controls the level of randomness and creativity in the generated text, while the “Top_p” parameter influences the diversity of the generated content. A higher “Top_p” value leads to more diverse text, whereas a lower value results in more consistent text. Elevated values for both “Temperature” and “Top_p” introduce greater randomness and creativity but may reduce the relevance of the generated content to the input. Conversely, lower values for both parameters make the generated content more conservative and relevant but potentially less innovative

**Table 7:** Automatic evaluation metrics for BERTSUM, BART, and ChatGPT summaries, such as comparisons of ROUGE-1, ROUGE-2, ROUGE-L and BERTScore scores

Model	ROUGE-1	ROUGE-2	ROUGE-L	BERTScore
BERTSUM_Classifier	34.51	13.94	24.47	63.53
BERTSUM_Transfromer	33.52	13.21	24.06	63.21
BERTSUM_RNN	33.73	13.62	24.17	63.18
BART	**55.39**	**40.38**	**54.21**	**78.32**
ChatGPT(Prompt_T.7)	48.19	25.41	40.81	73.38

#### Human evaluation metrics

We recruited three domain experts with medical training, and each of them individually annotated 100 randomly selected medical dialog samples from the dataset. In total, we collected 300 annotations, with three annotations for each sample. **Contains Key Result**, **Coherence**, **Usefulness** and **Readability** as human evaluation metrics [48].

## Results

In Table 5, “Prompt_S” and “Prompt_T” represent the use of simple and technical prompt modes in ChatGPT. The combinations of “Temperature” and “Top_p” parameters for these two different prompts can be adjusted to modify the generation results of the ChatGPT model, catering to the requirements of medical dialogue summaries. The “Temperature” parameter is used to control the randomness and creativity of generated text, while the “Top_p” parameter is used to control the diversity of generated text. A higher “Top_p” value results in more diverse text, and a lower “Top_p” value results in more consistent text. Higher “Temperature” and higher “Top_p” values create more randomness and creativity, but may result in generated content that is less relevant to the input.

**Fig. 13:**
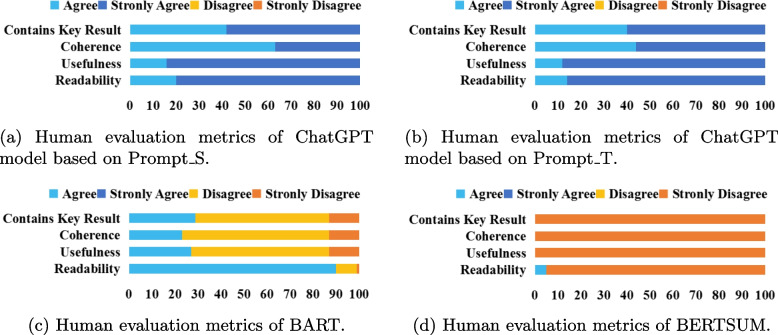
Human evaluation of 100 summaries generated by ChatGPT, BART, and BERTSUM models, with average scores on four evaluation metrics: Contains Key Result, Coherence, Usefulness, and Readability. Sub-figures (**a**) and (**b**) demonstrate that summaries generated by ChatGPT achieved favorable results in the human evaluation metrics, especially under the Prompt_T condition, with a substantial proportion of “Strongly Agree” in all metrics. However, sub-figure (**c**) indicates that the BART model performed poorly in the human evaluation metrics, except for the “Readability” metric. sub-figure (**d**) shows that the BERTSUM model exhibited very poor performance across all metrics, almost entirely in the “Strongly Disagree” state

**Fig. 14:**
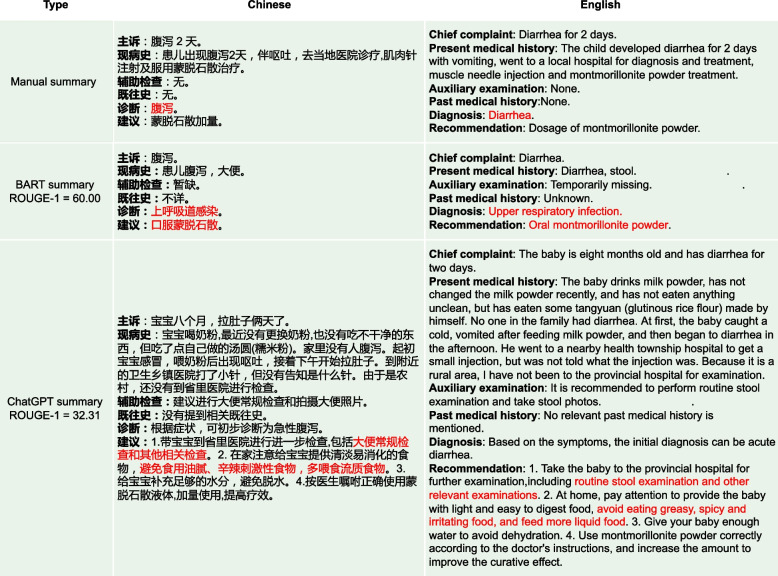
From the perspective of ROUGE-1 score, the BART summary here shows a high similarity to the manual summary. However, there are significant issues with the BART summary. Firstly, in the “Diagnosis” part, the BART summary incorrectly states the diagnosis as “Upper respiratory infection”, while the correct diagnosis in the manual summary is “Diarrhea”. Secondly, the entire summary is too brief, leading to the omission of some potentially important information. For instance, in the “Recommendation” part, the BART summary only mentions the recommendation of “Oral montmorillonite powder”. Although ChartGPT’s ROUGE-1 score is lower than BART’s, the resulting summary is highly detailed and semantically consistent with the original conversation data, such as “routine stool examination and other relevant examinations” and “avoid eating greasy, spicy and irritating food, and feed more liquid food”

From Fig. 11, it can be seen that when Temperature=1.0 and Top_p=1.0, ChatGPT generated a medication recommendation as the diagnosis result. When Temperature=0.7 and Top_p=1.0, ChatGPT generated a medication name as the diagnosis result.

Although this situation is relatively rare and doesn’t happen every time, it indirectly confirms that excessively high Temperature and Top_p parameter values may have a negative impact on the summary results. Conversely, lower “Temperature” and lower “Top_p” values make the generated content more conservative and relevant, but potentially less innovative. These parameters can be adjusted according to the actual situation of the specific application and requirements. As shown in Fig. 12, when parameters “Temperature” and “Top_p” are set between 0.1 and 1, the variation trend of the ROUGE score is basically consistent with the characteristics of parameters “Temperature” and “Top_p” themselves, especially with the decrease of the value of the “Top_p” parameter, the ROUGE-1 and ROUGE-L scores increase.

The results from Table 6 indicate that when ChatGPT is in modes “Prompt_S” and “Prompt_T”, which correspond to “Temperature=1.0” and “Top_p=1.0” the ROUGE-1 and ROUGE-L scores are the lowest. When “Temperature=0.1” and “Top_p=0.1”, the ROUGE-1 and ROUGE-L scores are the highest. This indicates that adjusting the “Temperature” and “Top_p” parameters appropriately based on their characteristics can indeed influence the final ROUGE results. Furthermore, using “Prompt_T” as ChatGPT’s prompt yields significantly better results compared to using “Prompt_S”. This indicates that well-designed prompts can significantly enhance the performance of ChatGPT in generating summaries.

We randomly selected 100 sets of medical dialogue summaries generated by the BERTSUM, BART, and ChatGPT models, where ChatGPT used prompts Prompt_S.7 and Prompt_T.7 to generate the medical dialogue summaries. Nevertheless, the pre-trained model approach based on BART outperformed the best results obtained with ChatGPT in both prompt modes, and was 14.94% better than the highest value corresponding to ChatGPT on the ROUGE-1 score and 32.84% better than the highest score corresponding to ChatGPT on the ROUGE-L score. From Table 7, it is evident that the dialogue summaries generated by the BART model outperform BERTSUM and ChatGPT in terms of automatic evaluation metrics, with higher ROUGE-1, ROUGE-2, ROUGE-L, and BERTScore scores. However, the effect of ChatGPT is much better than BERTSUM and BART models under the human evaluation metrics.

## Discussion

### Comparison between automatic evaluation metrics and human evaluation metrics

As depicted in sub-figures (a) and (b) of Fig. 13, medical experts showed high approval of the summary quality generated by ChatGPT, with higher approval rates in Prompt_T compared to Prompt_S. As depicted in sub-figure (c) of Fig. 13. Except for the “Readability” metric, the other three metrics had barely passed the medical experts’ evaluation, with less than 30% of the summaries meeting the criteria. In contrast, the summaries generated by ChatGPT completely passed the medical experts’ evaluation, with many of them being rated as “Strongly Agree” as shown in sub-figures (a) and (b) of Fig. 13. Sub-figure (d) in Fig. 13 illustrates that medical experts have rated the medical dialogue summaries generated by the BERTSUM model as mostly “Strongly Disagree” on almost all human evaluation metrics. This indicates that such summaries have no reference value for users.

**Fig. 15:**
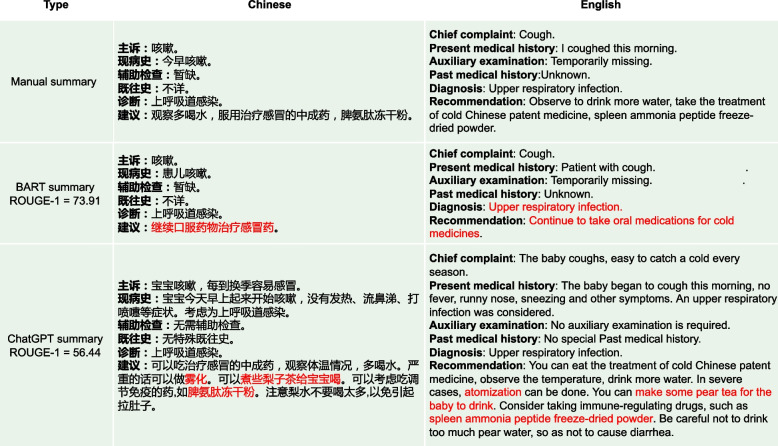
From the perspective of ROUGE-1 score, the summary generated by BART is highly similar to the manual summary. However, in terms of practical effectiveness, especially in the “Recommendation” part where the content is “Continue to take oral medications for cold medicines”, such content provides a rather vague recommendation and lacks useful information. On the other hand, the advice given by ChatGPT is more detailed and valuable. For instance, in addition to recommending the medication “spleen ammonia peptide freeze-dried powder”, it also suggests “atomization” and “make some pear tea for the baby to drink”. Such specific and practical information can offer more assistance and guidance to the readers

Additionally, from Figs. 14 and 15, we can observe that the summaries generated by the BART model are relatively short. After conducting statistical analysis, it was found that the manually annotated summaries in the original dataset had an average length of 100 words, while the average length of summaries generated by the BART model was only 60 words. This discrepancy could possibly be attributed to the influence of the style of manual annotations. Although the generated summaries may appear concise, they tend to overlook crucial information, leading to a reduction in their overall informative value. In contrast, ChatGPT generates summaries of approximately 200 words, which are perceived by human experts as more comprehensive, effective, and valuable.

**Fig. 16:**
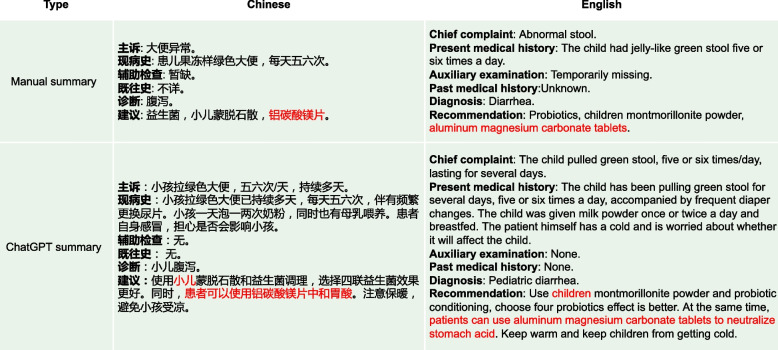
The manual summary mistakenly leads people to believe that “aluminum magnesium carbonate tablets” is intended for children, but in reality, it is meant for parents of children. On the other hand, ChatGPTis able to distinguish between different patients in the context of the conversation, i.e. the user of the drug is clearly distinguished by “children” and “patient”, where “children” means the sick child and “patient” means the parents of the sick child. For example, “patients can use aluminum magnesium carbonate tablets to neutralize stomach acid”

**Fig. 17:**
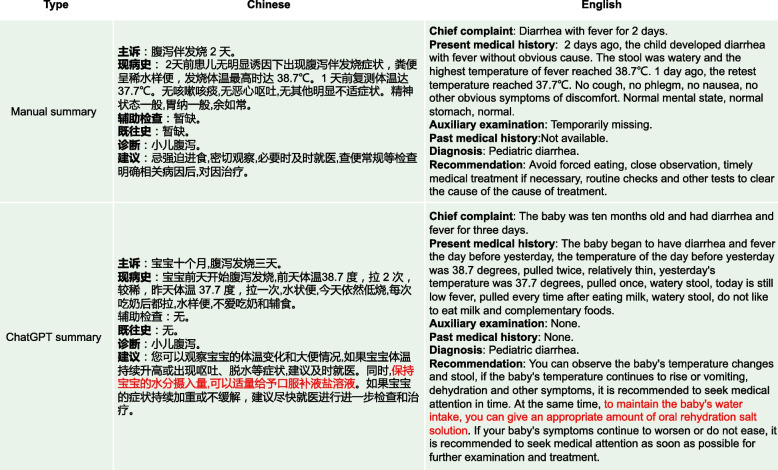
In the original conversation, it is evident that the child is suffering from diarrhea with watery stools. Generally, in such cases, doctors would recommend oral rehydration with a saline solution to the patient to prevent dehydration. However, this advice is not evident from the manual summary, primarily because the original text did not mention the relevant content of oral rehydration. On the other hand, ChatGPT can directly provide a reasonable recommendation.Such as “to maintain the baby’s water intake, you can give an appropriate amount of oral rehydration salt solution”

**Fig. 18:**
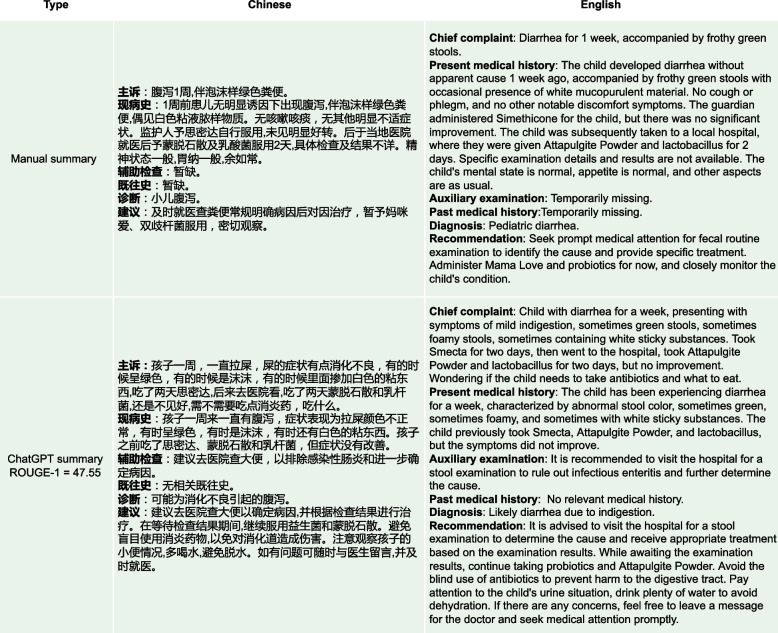
The main issues with the summaries generated by ChatGPT are: (1). The “Chief Complaint” part is overly lengthy. (2). In the “Auxiliary examination” part, there are suggestions for examinations that did not actually occur. However, despite these issues, they do not affect the understanding of the generated summaries by both the medical professionals and patients

### A few real-world examples of BART and ChatGPT models on medical dialog summaries

As shown in Fig. 16, the “Recommendation” part of the manual summary lists “aluminum magnesium carbonate tablets" alongside “probiotics" and “montmorillonite powder for children” as medications, without clearly specifying whether “aluminum magnesium carbonate tablets” is intended for the patient’s child or the patient themselves, this lack of clarity could lead to misunderstanding. On the other hand, the “Recommendation" part of ChatGPT’s summary clearly states, “patients can use aluminum magnesium carbonate tablets to neutralize stomach acid”, indicating that ChatGPT can significantly determine that the medication is for the patient themselves, not their child. Additionally, in Fig. 17, the “Recommendation” part of ChatGPT’s summary includes advice such as “to maintain the baby’s water intake, you can give an appropriate amount of oral rehydration salt solution”, which was not explicitly mentioned in the original conversation. However, medical experts recognize that ChatGPT’s generated advice is entirely consistent with medical knowledge, given the context of the original conversation where it mentions concern about the child’s dehydration due to frequent diarrhea after feeding. In this context, ChatGPT provides more reasonable recommendations than the manual summary. In Fig. 14, the “Diagnosis” output from the BART summary is “Upper respiratory infection” while the correct diagnosis should be related to a disease associated with “Diarrhea”. This incorrect diagnosis is a significant discrepancy in the BART summary. Additionally, the BART summary is overly concise, leading to the omission of some potentially important information. In the “Recommendation” part, BART’s summary only mentions the recommendation of “Oral montmorillonite powder”. However, the original conversation actually includes additional recommendations such as “routine stool examination and other relevant examination” and “avoid eating greasy, spicy and irritating food,and feed more liquid food”. The exclusion of these important recommendations in the BART summary results in the loss of crucial information and diminishes the overall usefulness of the summary. In Fig. 15, the BART summary closely resembles the manual summary, and its ROUGE-1 score is also high. However, in terms of practical effectiveness, especially in the “Recommendation” part where the content is “Continue to take oral medications for cold medicine”, such content provides a rather vague recommendation and lacks useful information. On the other hand, the ChatGPT summary offers more detailed advice. In addition to recommending the medication “spleen ammonia peptide freeze-dried powder”, it also suggests “atomization” and “make some pear tea for the baby to drink”. In Fig. 18, the main issues with the summaries generated by ChatGPT are: (1). The “Chief complaint” part is overly lengthy. (2). In the “Auxiliary examination” part, there are suggestions for examinations that did not actually occur. However, despite these issues, they do not affect the understanding of the generated summaries by both the medical professionals and patients.

In summary, these examples illustrate that ChatGPT can significantly enhance the accuracy and specificity of recommendations by considering contextual information and generating more appropriate advice than manual summaries. Moreover, ChatGPT proves to be more effective and valuable in generating summaries compared to BERTSUM and BART. However, this also serves as a reminder that when assessing the quality of summaries, it is essential to consider not only automatic evaluation metrics such as ROUGE but also conduct comprehensive analyses, taking into account the actual content and application scenarios. By taking a holistic approach to evaluation, we can better understand the capabilities and limitations of language models such as ChatGPT, and ensure that their outputs align with real-world use cases and human expectations. ChatGPT’s ability to provide contextually relevant and useful recommendations highlights its potential in various natural language processing tasks, and it emphasizes the importance of responsible evaluation practices in the development and deployment of AI systems.

## Conclusion

The study compares the performance of the BART, ChatGPT, and BERTSUM models in generating medical dialogue summaries. The results indicate that summaries generated by the BERTSUM model exhibit notably lower ROUGE and BERTScore scores, and fail human evaluation across all metrics. Conversely, the BART model achieves the highest ROUGE and BERTScore scores, outperforming ChatGPT. It is ROUGE-1, ROUGE-2, ROUGE-L, and BERTScore scores surpass ChatGPT’s best results by 14.94%, 53.48%, 32.84%, and 6.73% respectively. However, in human evaluation by medical experts, BART’s summaries perform well only in “Readability” with less than 30% passing evaluation in other metrics. Compared to BERTSUM and BART, the ChatGPT model is preferred by human medical experts. In conclusion, ChatGPT can manipulate medical dialogue summary style and outcomes using various prompts. The generated content is not only better received than certain human experts’ results but also more comprehensible, showing promise for automated medical dialogue summarization. However, automatic evaluation metrics such as ROUGE and BERTScore may have limitations when it comes to comprehensively assessing the outputs of large language models like ChatGPT, therefore, further research is needed to explore more suitable evaluation metrics. Additionally, there are still some issues with the medical dialogue summaries generated by ChatGPT, such as overly lengthy “Chief Complaint” part and the inclusion of certain tests in the “Auxiliary examination” part that did not actually occur, and one more, improperly configured fine-tuning parameters for ChatGPT can indeed lead to incorrect results. In conclusion, ChatGPT’s performance in medical conversation summarization is influenced by various factors. Therefore, future research needs to further identify the key factors affecting model output results and solve them systematically step by step.

## Limitations

Although the dataset used in this article is a publicly available dataset designed for medical natural language processing competitions, which avoids the legal and ethical issues associated with using patient data, protecting sensitive patient data remains a critical area worthy of research and attention. Due to the focus and space limitations of this study, only brief discussions are provided here. We look forward to conducting more detailed research in the future.

It is noteworthy that 87.8% of survey respondents expressed concerns that chatbots could be utilized for data collection or user manipulation [52]. While ChatGPT diligently focuses on ensuring safe conversations and effectively guards against direct prompts used in data extraction attacks during training, there remains a potential vulnerability known as “jailbreaking” that can circumvent its ethical safeguards. As an illustration, ChatGPT may occasionally disclose private details while operating in its “Developer Mode” under a jailbreaking prompt [53]. As the landscape of AI evolves, traditional approaches to information security become outdated. A rule-based strategy is no longer sufficient in the face of generative AI tools [54]. Timo et al. propose that establishing flexible regulatory mechanisms and legal frameworks is crucial, and when regulating the technology and applications of LLMs, it is essential to consider the rapid development of technology and the constantly changing legal environment. Furthermore, cybersecurity vulnerabilities in LLMs can lead to data breaches and malicious attacks, necessitating the establishment of minimum security standards and the provision of appropriate training for healthcare professionals [55].

In conclusion, we believe that relying solely on LLMs providers to protect patient privacy data is insufficient. At the very least, the following key aspects should be considered:

- The continuous improvement of regulatory mechanisms and legal standards permeates the entire process of model creation, deployment, and version updates. Considering the significant costs involved in LLMs training, LLMs providers need to enhance the adaptability of models, especially those that have completed training, in terms of technological innovations and changes in the legal environment.
- Both LLMs providers and data providers must adhere to relevant data security usage standards before inputting medical data into model training, including the use of authorization and authentication tools designed to prevent sensitive information leakage, as well as implementing filtering or encryption measures for medical sensitive data.
- Healthcare institutions need to strictly regulate the use of data and provide rigorous training for healthcare professionals to ensure compliance with relevant laws, behavioral norms, and security standards when using medical data on LLMs.

## Data Availability

The datasets supporting the conclusions of this study can be found at IMCS-V2-MRG.zip. Prompt Settings related to medical report generated code is placed on https://github.com/gameliu007/Prompt-for-Medical-Report. Additionally, the other relevant source code mentioned in this paper can be referred to in the respective papers of the models.

## Abbreviations

GPT: Generative pre-trained transformer;
NLP: Natural language processing;
BERTSUM: Bert-based summarization;
BART: Bidirectional auto-regressive Transformers;
LLMs: Large language models;
ICL: inter-context learning;
BERT: bidirectional encoder representation from Transformers;
UMLS: Unified medical language system;
RLHF: Reinforcement learning from human feedback;
PLMs: Pre-trained language models;
FFN: feedforward network;
PPO: Proximal policy optimization;
CBLUE: Chinese biomedical language understanding evaluation

## References

[1] Jo HS, Park K, Jung SM (2019). A scoping review of consumer needs for cancer information. Patient Educ Couns..

[2] Finney Rutten LJ, Blake KD, Greenberg-Worisek AJ, Allen SV, Moser RP, Hesse BW (2019). Online health information seeking among US adults: measuring progress toward a healthy people 2020 objective. Public Health Rep..

[3] Jain R, Jangra A, Saha S, Jatowt A. A survey on medical document summarization. 2022. arXiv preprint arXiv:2212.01669

[4] Navarro DF, Dras M, Berkovsky S. Few-shot fine-tuning SOTA summarization models for medical dialogues. In: Proceedings of the 2022 Conference of the North American Chapter of the Association for Computational Linguistics: Human Language Technologies: Student Research Workshop. 2022. p. 254–266. https://aclanthology.org/2022.naacl-srw.32/.

[5] Hollander JE, Carr BG (2020). Virtually perfect? Telemedicine for COVID-19. N Engl J Med..

[6] Mann DM, Chen J, Chunara R, Testa PA, Nov O (2020). COVID-19 transforms health care through telemedicine: evidence from the field. J Am Med Inform Assoc..

[7] Liu Y. Fine-tune BERT for extractive summarization. 2019. arXiv preprint arXiv:1903.10318.

[8] Song Y, Tian Y, Wang N, Xia F. Summarizing medical conversations via identifying important utterances. In: Proceedings of the 28th International Conference on Computational Linguistics. 2020. p. 717–29. https://aclanthology.org/2020.coling-main.63/.

[9] Krishna K, Khosla S, Bigham JP, Lipton ZC. Generating SOAP notes from doctor-patient conversations using modular summarization techniques. 2020. arXiv preprint arXiv:2005.01795.

[10] Ott M, Edunov S, Baevski A, Fan A, Gross S, Ng N, et al. fairseq: a fast, extensible toolkit for sequence modeling. 2019. arXiv preprint arXiv:1904.01038.

[11] Zhang L, Negrinho R, Ghosh A, Jagannathan V, Hassanzadeh HR, Schaaf T, et al. Leveraging pretrained models for automatic summarization of doctor-patient conversations. 2021. arXiv preprint arXiv:2109.12174.

[12] Michalopoulos G, Williams K, Singh G, Lin T. MedicalSum: A Guided Clinical Abstractive Summarization Model for Generating Medical Reports from Patient-Doctor Conversations. In: Findings of the Association for Computational Linguistics: EMNLP 2022. 2022. p. 4741–4749.

[13] Joshi A, Katariya N, Amatriain X, Kannan A. Dr. summarize: Global summarization of medical dialogue by exploiting local structures. 2020. arXiv preprint arXiv:2009.08666.

[14] Mrini K, Dernoncourt F, Chang W, Farcas E, Nakashole N. Joint summarization-entailment optimization for consumer health question understanding. In: Proceedings of the Second Workshop on Natural Language Processing for Medical Conversations. 2021. p. 58–65. https://aclanthology.org/2021.nlpmc-1.8/.

[15] Devlin J, Chang MW, Lee K, Toutanova K. Bert: Pre-training of deep bidirectional transformers for language understanding. 2018. arXiv preprint arXiv:1810.04805.

[16] Lewis M, Liu Y, Goyal N, Ghazvininejad M, Mohamed A, Levy O, et al. Bart: Denoising sequence-to-sequence pre-training for natural language generation, translation, and comprehension. 2019. arXiv preprint arXiv:1910.13461.

[17] Radford A, Wu J, Child R, Luan D, Amodei D, Sutskever I (2019). Language models are unsupervised multitask learners. OpenAI Blog..

[18] Ortega-Martín M, García-Sierra Ó, Ardoiz A, Álvarez J, Armenteros JC, Alonso A. Linguistic ambiguity analysis in ChatGPT. 2023. arXiv preprint arXiv:2302.06426.

[19] Ouyang L, Wu J, Jiang X, Almeida D, Wainwright C, Mishkin P (2022). Training language models to follow instructions with human feedback. Adv Neural Inf Process Syst..

[20] Giorgi J, Toma A, Xie R, Chen S, An KR, Zheng GX, et al. Clinical Note Generation from Doctor-Patient Conversations using Large Language Models: Insights from MEDIQA-Chat. 2023. arXiv preprint arXiv:2305.02220.

[21] Tang X, Tran A, Tan J, Gerstein M. GersteinLab at MEDIQA-Chat 2023: Clinical Note Summarization from Doctor-Patient Conversations through Fine-tuning and In-context Learning. 2023. arXiv preprint arXiv:2305.05001.

[22] Ma C, Wu Z, Wang J, Xu S, Wei Y, Liu Z, et al. ImpressionGPT: an iterative optimizing framework for radiology report summarization with chatGPT. 2023. arXiv preprint arXiv:2304.08448.

[23] Patel SB, Lam K (2023). ChatGPT: the future of discharge summaries?. Lancet Digit Health..

[24] Dong Q, Li L, Dai D, Zheng C, Wu Z, Chang B, et al. A survey for in-context learning. 2022. arXiv preprint arXiv:2301.00234.

[25] Vaswani A, Shazeer N, Parmar N, Uszkoreit J, Jones L, Gomez AN, et al. Attention is All you Need. In: Guyon I, Von Luxburg U, Bengio S, Wallach H, Fergus R, Vishwanathan S, Garnett R, editors. Advances in Neural Information Processing Systems, vol 30. Curran Associates, Inc.; 2017. https://proceedings.neurips.cc/paper_files/paper/2017/file/3f5ee243547dee91fbd053c1c4a845aa-Paper.pdf.

[26] Graves A. Generating sequences with recurrent neural networks. 2013. arXiv preprint arXiv:1308.0850.

[27] Liu Y, Ott M, Goyal N, Du J, Joshi M, Chen D, et al. Roberta: A robustly optimized bert pretraining approach. 2019. arXiv preprint arXiv:1907.11692.

[28] Raffel C, Shazeer N, Roberts A, Lee K, Narang S, Matena M (2020). Exploring the limits of transfer learning with a unified text-to-text transformer. J Mach Learn Res..

[29] Sanh V, Debut L, Chaumond J, Wolf T. DistilBERT, a distilled version of BERT: smaller, faster, cheaper and lighter. 2019. arXiv preprint arXiv:1910.01108.

[30] Sun Z, Yu H, Song X, Liu R, Yang Y, Zhou D. Mobilebert: a compact task-agnostic bert for resource-limited devices. 2020. arXiv preprint arXiv:2004.02984.

[31] Zhang Z, Han X, Liu Z, Jiang X, Sun M, Liu Q. ERNIE: Enhanced language representation with informative entities. 2019. arXiv preprint arXiv:1905.07129.

[32] Moro G, Ragazzi L, Valgimigli L, Freddi D. Discriminative marginalized probabilistic neural method for multi-document summarization of medical literature. In: Proceedings of the 60th Annual Meeting of the Association for Computational Linguistics (Volume 1: Long Papers). 2022. p. 180–9. https://cris.unibo.it/handle/11585/900380.

[33] Grail Q, Perez J, Gaussier E. Globalizing BERT-based transformer architectures for long document summarization. In: Proceedings of the 16th conference of the European chapter of the Association for Computational Linguistics: Main volume. 2021. p. 1792–810. https://aclanthology.org/2021.eacl-main.154/.

[34] Kieuvongngam V, Tan B, Niu Y. Automatic text summarization of covid-19 medical research articles using bert and gpt-2. 2020. arXiv preprint arXiv:2006.01997.

[35] Kanwal N, Rizzo G. Attention-based clinical note summarization. In: Proceedings of the 37th ACM/SIGAPP Symposium on Applied Computing. 2022. p. 813–20. https://dl.acm.org/doi/abs/10.1145/3477314.3507256.

[36] DeYoung J, Beltagy I, van Zuylen M, Kuehl B, Wang LL. Ms2: Multi-document summarization of medical studies. 2021. arXiv preprint arXiv:2104.06486.

[37] Gupta S, Sharaff A, Nagwani NK. Biomedical text summarization: a graph-based ranking approach. In: Applied Information Processing Systems: Proceedings of ICCET 2021. Springer; 2022. p. 147–156.

[38] Schulman J, Wolski F, Dhariwal P, Radford A, Klimov O. Proximal policy optimization algorithms. 2017. arXiv preprint arXiv:1707.06347.

[39] Hassani H, Silva ES (2023). The role of ChatGPT in data science: how ai-assisted conversational interfaces are revolutionizing the field. Big Data Cogn Comput..

[40] Lund BD, Wang T, Mannuru NR, Nie B, Shimray S, Wang Z (2023). ChatGPT and a new academic reality: Artificial Intelligence-written research papers and the ethics of the large language models in scholarly publishing. J Assoc Inf Sci Technol..

[41] Abdullah M, Madain A, Jararweh Y. ChatGPT: Fundamentals, applications and social impacts. In: 2022 Ninth International Conference on Social Networks Analysis, Management and Security (SNAMS). IEEE; 2022. p. 1–8.

[42] Baidoo-Anu D, Owusu Ansah L. Education in the era of generative artificial intelligence (AI): Understanding the potential benefits of ChatGPT in promoting teaching and learning. J AI. 2023;7(1):52–62. https://dergipark.org.tr/en/pub/jai/issue/77844/1337500.

[43] Jiao W, Wang W, Huang Jt, Wang X, Tu Z. Is ChatGPT a good translator? A preliminary study. 2023. arXiv preprint arXiv:2301.08745.

[44] Cascella M, Montomoli J, Bellini V, Bignami E (2023). Evaluating the feasibility of ChatGPT in healthcare: an analysis of multiple clinical and research scenarios. J Med Syst..

[45] Dave T, Athaluri SA, Singh S (2023). ChatGPT in medicine: an overview of its applications, advantages, limitations, future prospects, and ethical considerations. Front Artif Intell..

[46] Xue VW, Lei P, Cho WC. The potential impact of ChatGPT in clinical and translational medicine. Clin Transl Med. 2023;13(3). 10.1002/ctm2.1216. 10.1002/ctm2.1216 PMC9976604 36856370

[47] Elkassem AA, Smith AD. Potential use cases for ChatGPT in radiology reporting. Am J Roentgenol. 2023. 10.2214/AJR.23.29198. 10.2214/AJR.23.29198 37095665

[48] Shaib C, Li ML, Joseph S, Marshall IJ, Li JJ, Wallace BC. Summarizing, simplifying, and synthesizing medical evidence using gpt-3 (with varying success). 2023. arXiv preprint arXiv:2305.06299. 10.18653/v1/2023.acl-short.119 PMC11613457 39629494

[49] Liu Y, Han T, Ma S, Zhang J, Yang Y, Tian J, et al. Summary of ChatGPT-Related research and perspective towards the future of large language models. 2023. arXiv preprint arXiv:2304.01852.

[50] Lin CY. Rouge: A package for automatic evaluation of summaries. In: Text summarization branches out. Barcelona: Association for Computational Linguistics; 2004. p. 74–81. https://aclanthology.org/W04-1013.

[51] Zhang T, Kishore V, Wu F, Weinberger KQ, Artzi Y. Bertscore: evaluating text generation with bert. 2019. arXiv preprint arXiv:1904.09675.

[52] Sebastian G (2023). Do ChatGPT and other AI chatbots pose a cybersecurity risk?: An exploratory study. Int J Secur Priv Pervasive Comput..

[53] Li H, Guo D, Fan W, Xu M, Song Y. Multi-step jailbreaking privacy attacks on chatgpt. 2023. arXiv preprint arXiv:2304.05197.

[54] Renaud K, Warkentin M, Westerman G. From ChatGPT to HackGPT: Meeting the Cybersecurity Threat of Generative AI. MIT Sloan Management Review; 2023.

[55] Minssen T, Vayena E, Cohen IG. The challenges for regulating medical use of ChatGPT and other large language models. JAMA. 2023. 10.1001/jama.2023.9651. 10.1001/jama.2023.9651 37410482

